# JMM Profiles for the *Journal of Medical Microbiology*: an update

**DOI:** 10.1099/jmm.0.001515

**Published:** 2022-03-09

**Authors:** Norman K. Fry, Roberto M. La Ragione

**Affiliations:** ^1^​ Immunisation and Vaccine Preventable Diseases, UK Health Security Agency, London, UK; ^2^​ Vaccine Preventable Bacteria Section, Reference Services, Specialised Microbiology and Laboratories Directorate, UK Health Security Agency, London, UK; ^3^​ School of Biosciences and Medicine, Faculty of Health and Medical Sciences, Edward Jenner Building, University of Surrey, Guildford, UK; ^4^​ School of Veterinary Medicine, Faculty of Health and Medical Sciences, University of Surrey, Guildford, UK

**Keywords:** pathogen, antimicrobial, diagnostic, One Health, veterinary, *Actinobacillus pleuropneumoniae*

In March 2021 we announced the launch of a new article type for the *Journal of Medical Microbiology* (JMM), the ‘JMM Profile’ [1], with three specific categories – Pathogen Profile, Antimicrobial Profile and Diagnostic Profile – in order to provide brief summary reviews in each respective topic area.

Since the publication of our first Pathogen Profile on severe acute respiratory syndrome coronavirus 2 (SARS-CoV-2) by Professor Tim Inglis (JMM Deputy Editor-in-Chief) and Professor Kalai Mathee (JMM Co-Editor-in-Chief) [2], we have published a number of profiles, including those on *Vibrio cholerae *, *Bordetella pertussis* and *Streptococcus pneumoniae* [3–5]. We have also published our first Antimicrobial Profile on carbapenems [6], while our first Diagnostic Profile on loop-mediated isothermal amplification (LAMP) is forthcoming. In the next issues, we are publishing our first profiles focusing on veterinary pathogens, including one on avian paramyxovirus type-1 and Newcastle disease, and one on *Brachyspira* species.

In addition to medical and dental microbiology, JMM’s scope includes veterinary microbiology, which is concerned with microbial infections in domestic and wild animals. Domesticated animals, including livestock, farmed game, poultry and companion animals (including exotics), provide food or, indeed, other useful products or companionship to humans. Wild animals include those living in captivity (zoos, etc.) or as part of the natural fauna. Microbial diseases of animals are of interest and concern with regard to maintaining their overall health and in their interrelationship with humans and as a potential source of infection (zoonoses). Controlling infections in animals is also of significant interest with regard to food safety. The overuse of antimicrobials in livestock and companion animals and the subsequent contamination of the environment with antibiotics has often been cited as one of the main causes linked to the emergence and spread of antimicrobial resistance (AMR). Clearly, veterinary microbiology overlaps with medical microbiology and is a sentinel component of One Health.

Within JMM the ‘One Health – Emerging, Zoonotic and Environmental Diseases’ section encompasses the health of humans with animals and the environment, antimicrobial resistance, emerging zoonotic and environmental infectious diseases, veterinary, medical and comparative microbiology, together with understanding the epidemiology, surveillance and control of zoonotic pathogens from a medical microbiological perspective.

In this issue we are very pleased to publish our first veterinary Pathogen Profile focusing on an important bacterial disease of pigs: ‘* Actinobacillus pleuropneumoniae *: a major cause of lung disease in pigs, but difficult to control and eradicate’, by Professor Paul Langford and colleagues from Imperial College London [7]. *A. pleuropneumoniae* is an important respiratory pathogen of pigs that is responsible for significant economic and health and welfare issues in the pig industry. Therefore, a better understanding of the pathobiology of this important pathogen is urgently required to aid the development of novel mitigation strategies.

We thank you for your positive feedback to date on these profiles, and we look forward to your further suggestions for these and other JMM articles.
